# Natural Product-Derived Compounds for Targeting Multidrug Resistance in Cancer and Microorganisms

**DOI:** 10.3390/ijms241814321

**Published:** 2023-09-20

**Authors:** Maria-José U. Ferreira

**Affiliations:** Research Institute for Medicines (iMed.ULisboa), Faculty of Pharmacy, Universidade de Lisboa, Av. Gama Pinto, 1649-003 Lisbon, Portugal; mjuferreira@ff.ulisboa.pt

Natural products, characterized by huge scaffold diversity, complexity, and bioactivity, have long played a crucial role in drug discovery and development, particularly as anticancer and anti-infective agents. For millennia, natural products, biosynthesized by living organisms, have undergone an evolutionary process, acquiring specific properties for different biological functions, including mechanisms of defense and interaction with other organisms, thus justifying their role in cancer and infectious diseases. They cover a broad range of biologically important chemical space that cannot be explored by synthetic compounds. Moreover, in the case of medicinal plants, the ethnopharmacological information that is often available can provide insights into their safety and efficacy. In recent decades, the pharmaceutical industry has predominantly focused on synthetic compound libraries due to the challenges associated with natural products in the drug discovery process, namely isolation, characterization, and optimization. However, despite the challenges faced in natural products chemistry, the decrease in the number of new drugs that have entered the market, together with the recent scientific and technological advances, renewed interest in natural products, which remain a promising source of privileged bioactive structures, for direct use in clinics or as a starting material for the optimization of new drugs [1].

The development of drug resistance is the biggest threat to the successful treatment of cancer and infectious diseases.

Cancer is a leading cause of death globally. According to the World Health Organization (WHO), in 2020, it accounted for approximately 10 million deaths. The most common cancers include breast, lung, colon, and prostate cancers. Advancing age is the principal risk factor of cancer, where risk accumulation is combined with the less effective cellular repair mechanisms that occur with age. Despite the great enhancement of cancer treatment in recent decades, chemotherapy is still the standard cancer treatment. However, drug resistance is a major impairment in successful cancer chemotherapy in clinical practice. Ninety percent of deaths related to cancer are associated with treatment failure. Furthermore, cancer cells frequently exhibit cross-resistance to a variety of anticancer drugs that are not structurally related, which is termed the multidrug resistance (MDR) phenomenon and is the main cause of cancer relapse and cancer-related death. MDR is a complex and multifactorial phenomenon, which can result from several mechanism, such as an increase in drug efflux, alterations in drug targets or the metabolism of drugs, the enhancement of DNA damage repair, or a failure to undergo apoptosis. However, the most significant mechanism is the overexpression of transmembrane transporter proteins of the ATP-binding cassette (ABC) superfamily, which act as efflux pumps for chemotherapeutic agents, decreasing their intracellular concentration. Among them, P-glycoprotein (P-gp) is the most relevant drug transporter for MDR [2].

Likewise, the rise in antimicrobial resistance is one the major public health threats worldwide, making common infectious diseases difficult to treat or not treatable, and a leading cause of death, making the identification of new antimicrobial targets and/or new antimicrobial agents urgently needed. The number of bacterial infections caused by multidrug-resistant strains is increasing worldwide and WHO has identified antimicrobial resistance as a top ten global public health threat. The increased bacterial resistance to antibiotics currently in clinical use, together with the lack of new antibiotics in the market, make challenging to control drug-resistant infections. Bacteria can be intrinsically resistant to antibiotics or acquire resistance by different mechanisms, namely efflux of the antibiotic or low penetration into the bacterium, and alterations in the antibiotic target or inactivation of the antibiotic [3].

Thus, new strategies are imperative to address the problem of drug resistance in both cancer and infectious diseases, and many efforts have been carried out to face this issue. Although resistance can arise owing to multiple factors, drug efflux by transporter proteins is one of the major factors of the MDR phenotype.

The development of P-gp inhibitors that are able to reestablish drug sensitivity of resistant cells when co-administered with anticancer drugs has been considered a promising approach. Several P-gp inhibitors have reached clinical trials, but no inhibitors are clinically available. To solve this issue, many researchers are currently focused on natural products as a source of effective new MDR reversers [4].

Similarly, the overexpression of efflux pumps in bacteria can also confer resistance to antibiotics. While some efflux pumps have narrow substrate specificity, many are multidrug-resistance efflux pumps, transporting a wide range of structurally different substrates. Therefore, the development of efflux modulators to use in combination with antibiotics is considered a promising therapeutic strategy [5].

Drug combination studies have also been explored for tackling both cancer and bacterial resistance, aiming to achieve synergism. Due to synergistic effects, a combination of two or more drugs can improve their therapeutic benefits, allowing for the dose to be lowered, and thus reducing the emergence of drug resistance [5,6]. 

One other strategy for overcoming drug resistance in cancer and bacteria is the identification of collateral sensitivity agents. Cells and organisms that have developed resistance to one drug can simultaneously develop greater sensitivity to another drug, a concept known as collateral sensitivity. This effect can be used to select compounds that are highly effective against drug-resistant phenotypes [7,8].

Many natural products and derivatives, prepared by functionalization of natural products, have shown promising potential as resistance reversers in both resistant cancer cells and microorganisms.

This Special Issue, entitled “Natural Product-Derived Compounds for Targeting Multidrug Resistance in Cancer and Microorganisms”, includes six original articles focused on natural products or semisynthetic derivatives to tackle drug resistance in cancer and bacteria.

Schäfer et al. [9] screened, using in silico studies, an in-house library of 375 phytochemicals bearing diverse scaffolds for their ability to overcome multidrug resistance in cancer, and selected six compounds for further in vitro investigation. The six selected compounds were two flavonoids, one alkaloid, and three terpenes. They determined the growth-inhibitory effects towards wild-type leukemia cells and their resistant subline, overexpressing P-gp, and evaluated their ability as P-gp inhibitors in a functional assay. To obtain insights into the binding mode of these compounds, molecular docking studies were also carried out in wild-type and mutated P-gp forms in closed and open conformations. The authors emphasized the combination of in silico and in vitro techniques to select anti-MDR natural compounds and concluded that the flavonoid bidwillon A, isolated from *Erynthia sigmoidea*, and the terpene miltirone, from *Salvia miltiorrhiza*, are promising candidates for inhibiting wild-type and mutated P-glycoproteins and deserve further study.

Sun et al. [10] identified a methylated catechin derivative as a promising candidate for treating cancers overexpressing P-gp, in combination therapy. Tea polyphenols, such as epigallocatechin gallate, the most abundant catechin found in green tea, have low P-gp-modulating activity. To optimize their structures, Wong et al. prepared a library of catechin derivatives [11,12] and concluded that four methylated compounds were strong P-gp inhibitors. In the present work, these methylated catechin derivatives were further characterized by in vitro and in vivo studies, using sensitive and resistant human breast cancer cells, murine leukemia cells, and human leukemia cell lines as models. The results corroborated the previous studies. When compared with the starting compound, epigallocatechin gallate, which was not active, the authors concluded that the *O*-methylation of all rings and the linkers between ring D and C3 were essential for reversing P-gp-mediated MDR.

*Pancratium* species, included in the Amaryllidaceae family, can biosynthesize unique biologically active compounds, named Amaryllidaceae-type alkaloids, which are exclusive to the family and are characterized by strong pharmacological effects, including anticancer and anti-acetylcholinesterase properties. Aiming to find new P-gp-mediated MDR reversing compounds, the study of Sancha et al. [13] focused on the generation of a library of Amaryllidaceae alkaloids, by introducing new carbamate moieties into lycorine, a major alkaloid obtained isolated from the alkaloid fraction of the methanol extract of the bulbs of *Pancratium maritimum* [14]. Compounds were evaluated as MDR-reversers in resistant human colon adenocarcinoma cells overexpressing P-gp by functional and chemosensitivity assays. Compared to lycorine, most of the compounds showed higher P-gp-inhibitory activity. The strongest inhibitors were the di-carbamate derivatives bearing phenethyl or benzyl moieties. Furthermore, some compounds revealed a collateral sensitivity effect and interacted synergistically with doxorubicin. Selected compounds behaved as inhibitors in the ATPase activity. 

Curcumin is a polyphenol found in the rhizome of *Curcuma longa* Linn (turmeric). It has long been used in traditional medicine due to its beneficial effects and is the major ingredient of the spice curry. Curcumin can interact with diverse cellular targets, being well-known for its multiple biological activities, namely anti-oxidant, anti-inflammatory, and anticancer properties. There have been numerous studies reporting the in vitro anticancer properties of curcumin in several cancer cells. In vivo studies were also reported, as well as human clinical trials. It mainly acts by inducting apoptosis or sensitizing cancer cells to other anticancer agents. The aim of the work of Wu et al. [15] was to investigate the cytotoxic activity of curcumin in chemoresistant lung cancer cells with different P-gp expression levels. The mitogen-activated protein kinase signaling pathway and the role of endoplasmic reticulum (ER) stress, induced by reactive oxygen species, were also addressed. 

Jia et al. [16] reported that a brominated derivative of jelleine-I, a peptide found in the royal jelly of honeybees, exhibited potent antibacterial activity against *Fusobacterium nucleatum*, a gram-negative bacterium that has been associated with colorectal cancer. According to WHO, colorectal cancer is the third most common cancer and the second leading cause of cancer-related deaths worldwide. It is frequently diagnosed in advanced stages, with limited treatment options, and there is an urgent need for effective treatment. It is associated with several lifestyle factors, namely an inactive lifestyle, obesity, smoking, alcohol consumption and dietary habits. Moreover, there is also increasing evidence for the association of colorectal cancer with the intestinal microbiota, which plays a crucial role in human health. In fact, an imbalance in intestinal microbiota can cause several intestinal diseases, including cancer. According to the authors, bromine-jelleine-I could be considered as an adjunctive agent for colorectal cancer treatment. 

Methicillin-resistant *Staphylococcus aureus* (MRSA) is one of the leading causes of hospital-acquired infections, responsible for high infection and mortality rates, and there is an urgent need for new treatment strategies. Aiming at developing alternatives to antibiotics for the treatment of multi-drug-resistant *Staphylococcus aureus* infections, Liu at al [17] reported the isolation from patients and environmental sources, and characterization, of four lytic anti-staphylococcal bacteriophages. Bacteriophages, known as phages, are natural antimicrobials that exist everywhere in nature. They are viruses that specifically infect bacteria and are safe for humans, animals, plants, and the environment. They were discovered more than 100 years ago. Phage therapy for bacterial infections has long been used in both human and veterinary medicine and in agriculture-related products. However, with the development of antibiotics, the use of phage therapy to treat bacterial infections decreased or was abandoned. Lately, due to the increase in antibiotic resistance, and the recent clinical success with phage cocktails, phage therapy has been attracting renewed interest. It is considered that, despite the challenges being faced, phage therapy has great potential to treat antibiotic-resistant bacterial infections. 

In summary, the works reported in this Special Issue provide evidence for the importance of natural products to find out new agents to overcome multidrug resistance. 
